# Susceptibility to COPD: Differential Proteomic Profiling after Acute Smoking

**DOI:** 10.1371/journal.pone.0102037

**Published:** 2014-07-18

**Authors:** Lorenza Franciosi, Dirkje S. Postma, Maarten van den Berge, Natalia Govorukhina, Peter L. Horvatovich, Fabrizia Fusetti, Bert Poolman, Monique E. Lodewijk, Wim Timens, Rainer Bischoff, Nick H. T. ten Hacken

**Affiliations:** 1 University of Groningen, Department of Pharmacy, Analytical Biochemistry, Groningen, The Netherlands; 2 University of Groningen, University Medical Centre Groningen, Department of Pulmonary Diseases, Groningen Research Institute of Asthma and COPD (GRIAC), Groningen, The Netherlands; 3 Department of Biochemistry, University of Groningen, Netherlands Proteomics Centre, Groningen, The Netherlands; 4 University of Groningen, University Medical Centre Groningen, Department of Pathology, Groningen Research Institute of Asthma and COPD (GRIAC), Groningen, The Netherlands; The University of Auckland, New Zealand

## Abstract

Cigarette smoking is the main risk factor for COPD (Chronic Obstructive Pulmonary Disease), yet only a subset of smokers develops COPD. Family members of patients with severe early-onset COPD have an increased risk to develop COPD and are therefore defined as “susceptible individuals”. Here we perform unbiased analyses of proteomic profiles to assess how “susceptible individuals” differ from age-matched “non-susceptible individuals” in response to cigarette smoking. Epithelial lining fluid **(ELF)** was collected at baseline and 24 hours after smoking 3 cigarettes in young individuals susceptible or non-susceptible to develop COPD and older subjects with established COPD. Controls at baseline were older healthy smoking and non-smoking individuals. Five samples per group were pooled and analysed by stable isotope labelling (iTRAQ) in duplicate. Six proteins were selected and validated by ELISA or immunohistochemistry. After smoking, 23 proteins increased or decreased in young susceptible individuals, 7 in young non-susceptible individuals, and 13 in COPD in the first experiment; 23 proteins increased or decreased in young susceptible individuals, 32 in young non-susceptible individuals, and 11 in COPD in the second experiment. SerpinB3 and Uteroglobin decreased after acute smoke exposure in young non-susceptible individuals exclusively, whereas Peroxiredoxin I, S100A9, S100A8, ALDH3A1 (Aldehyde dehydrogenase 3A1) decreased both in young susceptible and non-susceptible individuals, changes being significantly different between groups for Uteroglobin with iTRAQ and for Serpin B3 with iTRAQ and ELISA measures. Peroxiredoxin I, SerpinB3 and ALDH3A1 increased in COPD patients after smoking. We conclude that smoking induces a differential protein response in ELF of susceptible and non-susceptible young individuals, which differs from patients with established COPD. This is the first study applying unbiased proteomic profiling to unravel the underlying mechanisms that induce COPD. Our data suggest that SerpinB3 and Uteroglobin could be interesting proteins in understanding the processes leading to COPD.

## Introduction

Chronic obstructive pulmonary disease (COPD) is a major leading chronic disease and the only one with increasing prevalence and mortality worldwide. It is characterized by chronic, progressive airflow limitation [1]. The pathology of COPD includes a complex network of inflammation, oxidative stress, tissue damage, remodelling and repair [2]. It comprises many detrimental processes that contribute to disease progression, a progression that is relentless and without a cure. Further research in this area is thus important, since a better understanding of COPD pathogenesis will enable the development of new and more effective treatments for the prevention and progression of COPD. Proteomics is an emerging scientific research field with important advances in proteomic instrumentation and methodology leading to the possibility to identify in small quantities of biological material an entire set of proteins important for the pathophysiology of a complex disease like COPD [3]. In COPD a relative low number of proteomic studies has been performed [3], using different methods [4], [5], in biological materials like bronchoalveolar fluid (BALF) [6]–[9], induced sputum [10]–[13] and exhaled breath condensate [14]. Although promising disease-specific and severity-related biomarkers came out [4], not one study focused on the very first phase of the induction of COPD.

In the past, investigating the acute response to cigarette smoking has been put forward as an attractive approach to understand the pathogenesis of COPD [15], [16]. This so called acute smoking model is attractive because inflammatory responses of the lung to cigarette smoke can be investigated in a standardised and dynamic way. Although highly standardised, the acute smoking results in human studies demonstrate remarkably high inter-individual differences to cigarette smoking [16], [17]. This variation may be due to methodological issues of assessing inflammatory responses, however, it could also reflect a really different response between individuals. In this perspective, it is important to acknowledge that only 20–30% of the smokers develop COPD, suggesting that a specific genetic background plays a role in the pathogenesis of COPD [18]. Indeed previous studies have suggested that family members of patients with severe early-onset COPD have an increased risk to develop COPD with smoking [19], and can therefore be labelled as “susceptible individuals”. Thus far the mechanisms that lead to development of COPD in susceptible smokers remain largely unknown.

In this study we hypothesize that the acute smoking model is an attractive tool to better understand the essential differences between susceptible and non-susceptible individuals. This will especially be important in young individuals with a low number of pack-years smoking since they still have clean and uncompromised lungs. In other words, we hypothesize that ageing and lifelong smoking leads to altered airways not reflecting the very first aberrant response to cigarette smoking at young age. For this reason, we set out to investigate the onset of COPD in an acute smoking experiment in young healthy individuals, being susceptible” or “non-susceptible” to develop COPD. To address this point, we profiled proteins in epithelial lining fluid (ELF), prior to and 24 h after a controlled smoking episode in susceptible and non-susceptible young individuals. In addition, we compare these results with those in older subjects with established COPD. We chose to investigate ELF because this biologically active fluid constitutes the very first barrier to cigarette smoke, and because proteomic analysis of undiluted ELF recovered by a bronchoscopic microsampling probe contains many proteins associated with lung disease [20].

## Materials and Methods

### Subjects

This study was part of a larger multi-centre study (www.clinicaltrials.gov, NCT00807469) [21]. Subjects were recruited at the outdoor clinic of the University Medical Centre Groningen (UMCG). Young (18–45 years) subjects were divided in those who were susceptible or not susceptible to develop COPD. Susceptibility was based on family history: not susceptible refers to subjects with smoking family members who are at least 45 years old yet without having COPD. Susceptible individuals needed to have a high prevalence of COPD in smoking family members older than 45 years: 2 out of 2, 2 out of 3, 3 out of 3, 3 out of 4, or 4 out of 4. All subjects were “party smokers” with <10 pack-years smoking, who were able to stop smoking for at least two days and start smoking on request. Old (>45 years) subjects with established COPD (GOLD II) and > 10 pack-years smoking were included for comparison. In addition, two control groups of old individuals were included: subjects with normal lung function despite > 10 pack/years smoking (healthy smokers), and subjects with normal lung function and no smoking history (healthy non-smokers).

The study was approved by the Medisch Ethische Commissie Universitair Medisch Centrum Groningen (METc 2008–136), and all subjects gave their written informed consent.

### Smoking, Elf Collection and Sample Preparation

Young susceptible and non-susceptible individuals and old COPD patients participated in the acute smoking experiments. The healthy smoking and non-smoking individuals did not perform smoking experiments and served as controls for COPD patients at baseline. All subjects were not allowed to smoke for at least two days prior to the experiments. Immediately before smoking exhaled CO was measured to ascertain that individuals had not smoked recently, and immediately after smoking to confirm that all individuals inhaled cigarette smoke sufficiently. If subjects had an exhaled CO >5 ppm, indicating recent cigarette smoking, they were not allowed to participate in the acute smoking experiment. In the acute smoking experiment, all subjects smoked 3 Marlboro cigarettes within one hour under supervision; always at the same time of the day between 9 and 11 A.M. Data from subjects who did not inhale sufficiently (exhaled CO <2 ppm) was not included. Bronchoscopy was performed both 24 hours after smoking and 6 weeks later in a stable phase to obtain baseline data. All bronchoscopies were carried out according to international guidelines [22]. ELF was collected at the mucosa of the left main bronchus using 3 microsampling probes (BC-401C; Olympus, Tokyo, Japan) [23].

### Stable Isotope Labelling

ELF samples containing 50 µg total protein were used for iTRAQ labelling. The procedure was performed as previously described [24], [25]. Briefly, each tryptic digested sample was labelled (iTRAQ Reagent 4-plex, ABSciex, Foster City, CA, USA) according to the manufacturer's protocol. The individually labelled digests were then combined into a single sample mixture and subjected to strong-cation exchange chromatography (AKTA Purifier, GE Healthcare Biosciences AB, Uppsala, Sweden). The resulting peptide-containing fractions were separated by reversed-phase chromatography (Ultimate 3000 nanoflow liquid chromatography system, Dionex, Amsterdam, The Netherlands). Fractions of 12 sec were spotted on MALDI targets (Probot, Dionex, Amsterdam, The Netherlands) and mass spectrometric analysis was carried out on a 4800 Proteomics Analyzer MALDI TOF/TOF instrument (Applied Biosystems, Foster City, CA, USA) controlled by the 4000 Series Explorer v3.5 software.

**Figure 1:**
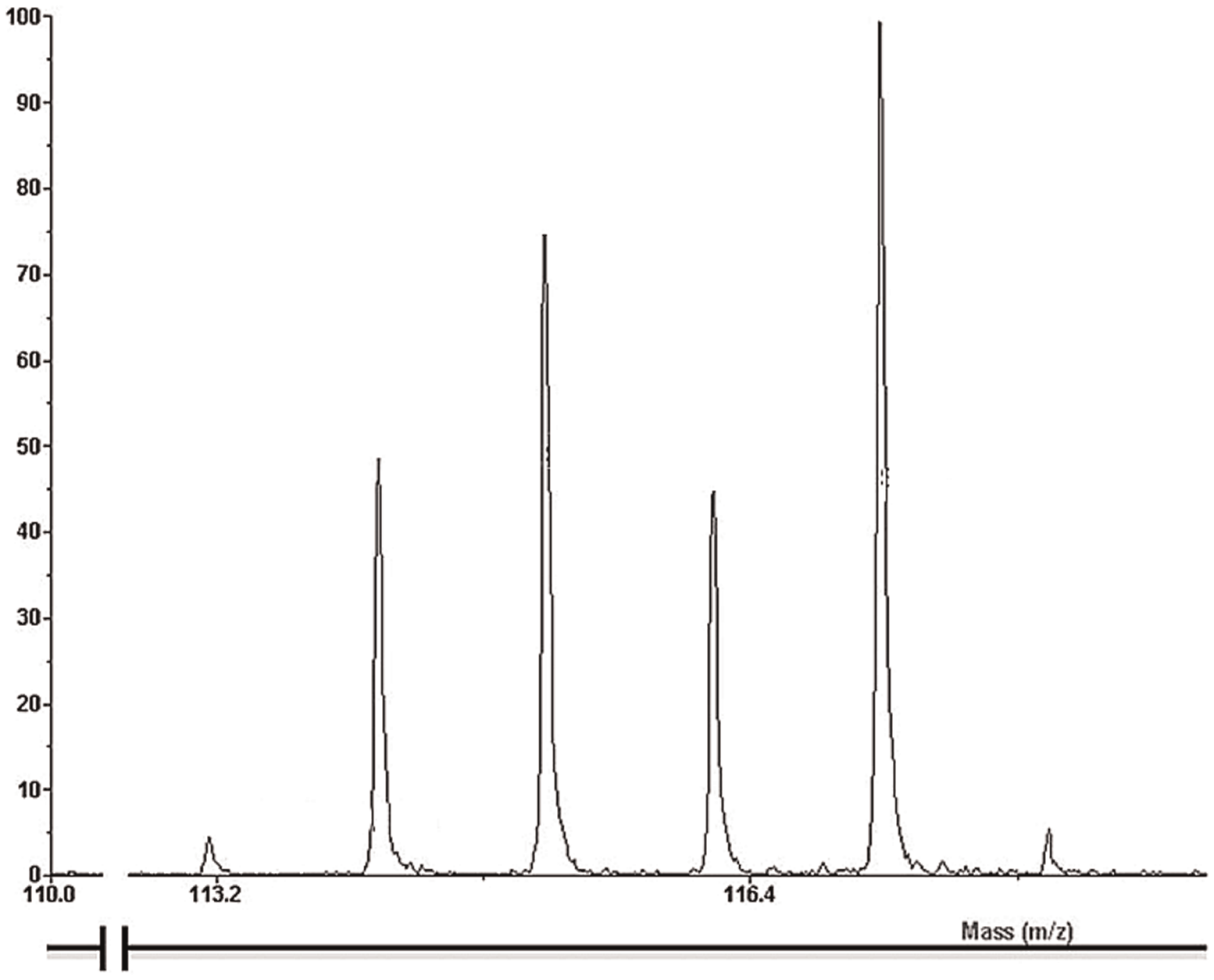
Reporter ion pattern of Peroxiredoxin I (peptide LVQAFQFTDK). Peaks at 114, 115, 116 and 117 represent the group of young non-susceptible after acute smoking; young non-susceptible at baseline, young susceptible after acute smoking and young susceptible at baseline, respectively.

Proteins were identified using Protein Pilot software v4.0 (Applied Biosystems). The identification was performed using the IPI Human database (IPI v3.83). The Protein Pilot cut-off score was 1.3, corresponding to a confidence limit of 95% at the peptide level. Protein identifications were based on at least 2 unique peptides identified independently. A probability higher than 95% and a false discovery rate lower than 5%, were accepted. The experiments were repeated with the same set of samples. ProQuant software was used to calculate the intensity of 3 reporter ions (m/z: 115, 116 and 117, **Figure 1**) and to divide them by the intensity of the 4^th^ reporter ion (m/z: 114) for each measured compound. All ratios were transformed into natural logarithms and plotted against the number of peptides subjected to MS/MS analysis. Gaussian curves were fitted on the smoothed histograms (histogram between −1 and +1 with 200 steps, smoothed using a Savitzky-Golay algorithm) and standard deviations (SD) were determined. Proteins with natural log-transformed ion ratios differing by at least 2.5×SD (98.8% confidence) were considered significantly different from the random variation. Visual explanation of the applied method is presented in **Figure S1 in the File S1**. All data pre-processing work was done on a personal computer equipped with a +3600 MHz AMD processor and 4 GB of RAM, using MATLAB 7.11.0.584 (R2010b).

### ELISA

Due to methodological problems with the commercially available ELISA kits, we were unable to obtain results for S100A8 and ALDH3A1. The other four selected proteins were all above the detection limit of the ELISA. Commercially available ELISA kits from Uscn Life Science Inc. (China) were used following the manufacturer's protocols. Briefly, 100 uL of undiluted ELF were incubated for 2 hours at 37°C in microtitre plates precoated with the specific monoclonal antibody. Subsequently a biotin-conjugated polyclonal antibody was added, followed by a TMB substrate solution and finally the reaction was stopped adding 50 uL of a sulphuric acid solution. The absorbance of each sample and calibration curve was read at 450 nm. The protein concentration in the samples was determined comparing the absorbance values of the samples to the standard curve.

Statistical analyses were performed using SPSS (version 16.0; SPSS, Chicago IL). Baseline differences between young non-susceptible versus young susceptible individuals and between old healthy smokers versus non-smokers and COPD were tested using Mann-Whitney U tests. Changes associated with smoke exposure (before and after acute exposure to cigarette smoke) within the group of young non-susceptible individuals, young susceptible individuals and COPD patients were tested using Wilcoxon tests. P-values < 0.05 were considered statistically significant.

### Immunohistochemistry

Immunohistochemistry of Aldehyde dehydrogenase 3A1 was performed to compare lung tissue from COPD patients who underwent lung transplantation (5 current smokers and 5 ex-smokers) and non-COPD controls who underwent surgery for lung cancer (5 current smokers and 5 never/ex-smokers). Three-µm thick lung sections were cut from selected formalin-fixed paraffin-embedded tissue blocks; immunostaining and quantification was performed as previously described [26]. Anti-ALDH3A1, SAB1405446 (Sigma-Aldrich Chemie BV, Zwijndrecht, The Netherlands) was used as primary antibody. Sections were scored semi-quantitatively.

## Results

### Subjects

A total of 25 subjects were selected for the iTRAQ experiments, 5 participants per group (**Table 1** **upper section**). There was no significant difference in the clinical characteristics between the young susceptible and non-susceptible subjects, although there was a trend for higher age in the former group (p = 0.16). COPD patients had a higher age than the old healthy smokers and non-smokers (p = 0.009 and p = 0.006, respectively). The COPD patients demonstrated airway obstruction compatible with GOLD stage II and 6 (out of 8) subjects demonstrated signs of emphysema (CO diffusion < 80% predicted). To verify the proteins detected by iTRAQ, eighteen additional subjects divided over the above groups were additionally included to enhance the numbers in the ELISA experiments, resulting in a total of 43 participating subjects (**Table 1** **lower section**).

**Table 1:** Characteristics of the participating subjects.

	Acute smoking experiment			Baseline controls	
	Young healthy susceptible	Young healthy non-susceptible	Old COPD	Old healthy smokers	Old healthy never-smokers
***A. Subjects participating in the iTRAQ study***					
Male/Female, n	3/2	0/5	0/5	3/2	1/4
Age, years	29 (18–42)	20 (19–39)	66 (55–74)	50 (47–53)	49 (45–53)
Pack years, n	0 (0–8)	2 (0–9)	23 (21–46)	38 (11–52)	0
FEV_1_, % pred	103 (97–108)	109 (98–117)	74 (49–80)	111 (105–32)	111 (109–122)
FEV_1_/FVC, %	80 (76–94)	81 (77–91)	54 (32–60)	80 (74–85)	76 (75–82)
TLC, % pred	25 (23–28)	22 (16–25)	39 (38–55)	36 (32–37)	33 (31–36)
CO diffusion, mmol/min/kPa	84 (80–97)	87 (62–98)	71 (40–86)	84 (74–96)	106 (84–117)
***B. Subjects participating in the ELISA study***					
Male/Female,n	3/4	0/6	0/8	6/3	8/5
Age, years	29 (18–42)	21 (19–39)	66 (55–74)	54 (47–70)	54.5 (45–70)
Pack years, n	0 (0–8)	2 (0–9)	28 (20–49)	39 (11–52)	0
FEV_1_, % pred	108 (100–116)	109 (98–117)	68 (49–80)	110 (101–121)	111 (93–122)
FEV_1_/FVC, %	78 (76–94)	82 (77–91)	52 (32–60)	78 (70–85)	78 (74–82)
TLC, % pred	25 (23–28)	22 (16–25)	38.5 (33–55)	36 (32–41)	36 (31–43)
CO diffusion, mmol/min/kPa	85 (84–102)	88 (62–98)	64 (40–91)	88 (83–117)	106 (84–119)

Values are medians (ranges) or numbers.

### Proteomics

#### General results

Pooled ELF samples (n = 5 per group) labelled with stable isotopes (iTRAQ4-plex) were analysed by mass spectrometry in duplicate (**Table S1–S2 in File S1**). In the group of young subjects 64 overlapping proteins were identified; in the older group 70 proteins (**Figure S2 in File S1**). At baseline, 6 overlapping proteins were differentially expressed between young susceptible and young non-susceptible individuals; and 7 between old healthy smokers and never-smokers (**Table S3 and figure S3 in File S1**). After acute smoking of 3 cigarettes, the number of differentially expressed proteins showing overlap between the first and second experiment was 9 proteins in the group of the young susceptible individuals, 3 in the young non-susceptible individuals and 3 in the COPD patients (**Table S4 and figure S4 in File S1**).

The complete list of all proteins and relative peptides identified and quantified with high confidence (>95%) is reported in **Table Data S1**.

#### Proteomics: selection of differential proteins

The following proteins were selected for further analysis with ELISA or immunohistochemistry based on the following criteria: 1) significant up- or down-regulation in both iTRAQ experiments, 2) quantification with 2 or more statistically significantly different peptides (p value < 0.02), 3) biological function that might be implicated in the onset and progression of COPD.

- Peroxiredoxin I (accession number Q06830),
- Uteroglobin (CC16, Clara Cell 16, accession number P11684),
- SerpinB3 (SCCA1, accession number P29508),
- S100A8 (MRP8, Calgranulin A, accession number P05109),
- S100A9 (MRP14, Calgranulin B, accession number P06702),
- Aldehyde dehydrogenase 3A1 (ALDH3A1, accession number P30838).

#### Proteomics: comparison between groups at baseline

There were no significant differences in Peroxiredoxin I, Uteroglobin and ALDH3A1, between young susceptible and young non-susceptible individuals, while SerpinB3, S100A9, and S100A8 levels were higher in the young susceptible group (**Table 2**). Old healthy smokers showed higher levels of ALDH3A1 and Peroxiredoxin I than old healthy non-smokers.

**Table 2:** Summary of iTRAQ comparisons from pooled ELF samples.

	Acute smoking comparisons			Group comparisons at baseline	
	Young healthy susceptible	Young healthy Non-susceptible	Old COPD	Young healthy susceptible vs non-susceptible	Old healthy smokers vs never-smokers
**Peroxiredoxin I**	**0.29**	**0.50**	**6.9**	σ <2.5	σ <2.5
**Uteroglobin**	σ <2.5	**0.50**	**0.1**	σ <2.5	σ <2.5
**SerpinB3**	σ <2.5	**0.40**	11.6*	**2.42**	σ <2.5
**S100A9**	**0.39**	**0.50**	σ <2.5	**3.51**	σ <2.5
**S100A8**	**0.35**	**0.46**	σ < 2.5	**2.93**	σ <2.5
**ALDH3A1**	**0.29**	**0.29**	**16.7**	σ <2.5	**7.80**

Data are expressed as median of ratios (of peptides for one protein that are discriminatory between samples): after smoking/before smoking (left section) or group comparisons (right section). σ <2.5: peptides of that protein did not reach a statistically significant difference. *: based on one peptide.

#### Proteomics: comparison before and after acute smoking

In the young susceptible individuals levels of Peroxiredoxin I, S100A9, S100A8 and ALDH3A1 decreased after acute smoke exposure (**Table 2**) while all selected proteins were down-regulated in the young non-susceptible group. On the contrary, Peroxiredoxin I, SerpinB3, and ALDH3A1 were up-regulated in the old COPD patients, whereas Uteroglobin was down-regulated after acute smoke exposure (**Table 2**).

#### ELISA: comparison between groups at baseline

Young susceptible individuals showed a trend for lower SerpinB3 concentrations in ELF than young non-susceptible individuals (p = 0.056).There were no significant differences between old healthy smokers versus non-smokers, nor between COPD patients and the two old healthy groups.

#### ELISA: comparison before and after acute smoking

**Figure 2:**
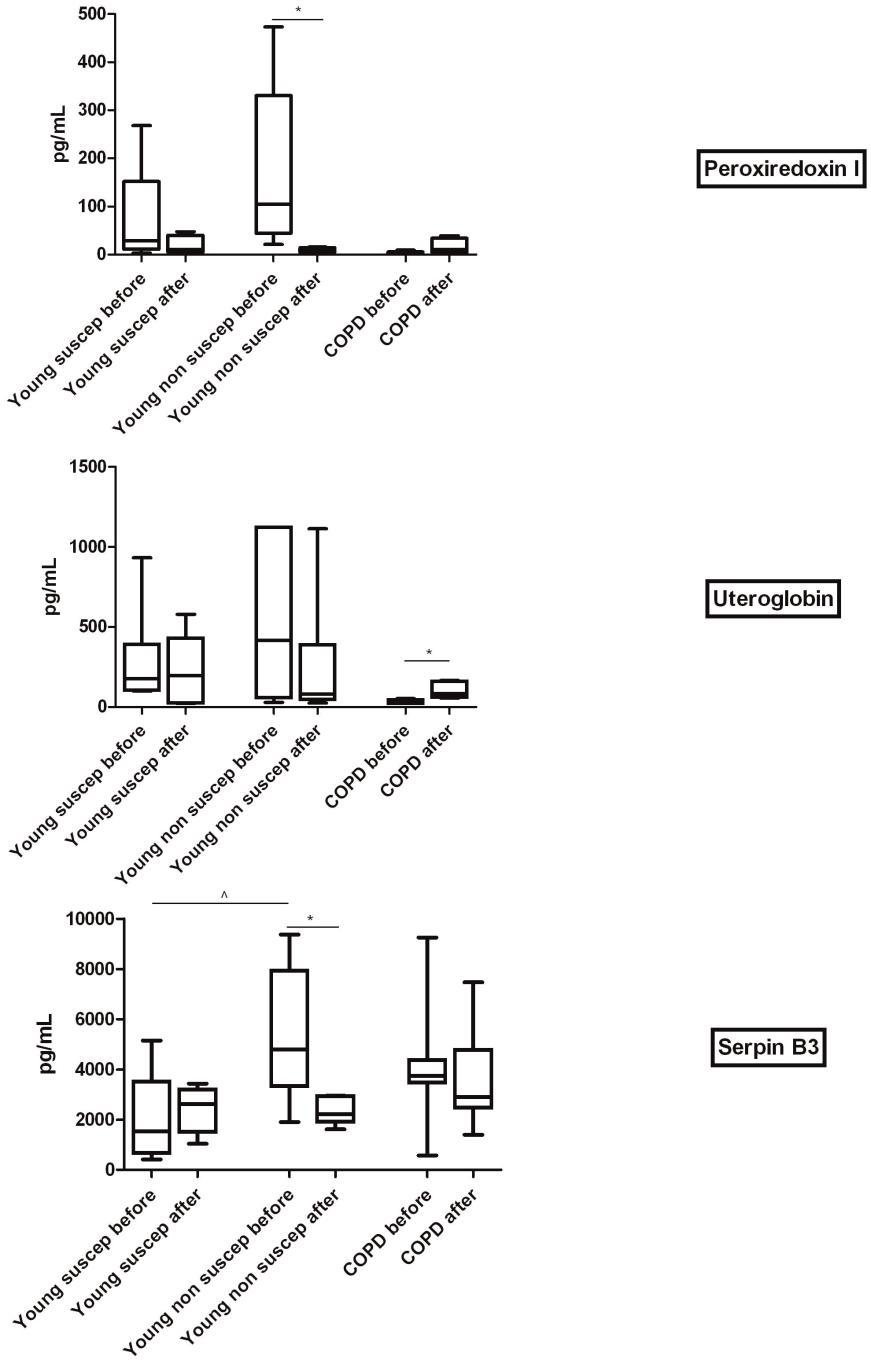
ELISA results of individual epithelial lining fluid (ELF) samples of young susceptible individuals, young non-susceptible individuals, and established COPD patients, before and after acute smoking. Results are given in box-plots with medians and interquartile ranges. *: p<0.05 before vs after smoking, ∧: p<0.05 vs young susceptible individuals at baseline.

In young susceptible individuals, expression of the selected proteins did not differ significantly before and after acute smoking (**Table 3**). In the young non-susceptible individuals Peroxiredoxin I and S100A9 concentrations were lower after smoking (p = 0.043, and p = 0.028, respectively), whereas SerpinB3 showed a similar trend (p = 0.08) (**Figure 2**).The comparison between young non-susceptible and susceptible individuals regarding their acute smoking response showed a significant difference in the change of SerpinB3 with smoking (**Table 3**, Mann Whitney U test, p = 0.016). In the COPD patients Peroxiredoxin I tended to increase after acute smoking (p = 0.063).

**Table 3:** ELISA results of non-pooled ELF.

	Acute smoking experiment			Baseline controls	
	Young Susceptible	Young NON-susceptible	Old COPD	Old healthy smokers	Old healthy never-smokers
Peroxiredoxin I, pg/mL Before	28.5 (3.3–268)	105 (21–473)	3.8 (0.4–8.8)	36.5 (1.8–227)	13 (1.1–164)
Peroxiredoxin I, pg/mL After	10.5 (0.07–48)	10.5 (1.9–15.5)*	10.8 (2.4–39)∧		
Uteroglobin, pg/mL Before	176 (100–933)	415 (29–1123)	24 (21–52)	409 (17–1484)	134 (29–764)
Uteroglobin, pg/mL After	195 (21–580)	81 (25–1115)	84 (54–166)		
Serpin B3, pg/mL Before	**1536 (417–5152)#**	**4803 (1900–9371)**	3745 (567–9254)	3935 (798–4454)	2476 (821–4904)
Serpin B3, pg/mL After	**2609 (1040–3439)**	**2210 (1610–2955)**	2907 (1398–7474)		
S100 A9, µg/mL Before	0.24 (0.01–0.96)	0.43 (0.17–2.80)	0.63 (0.37–0.87)	0.9 (0.2–5.2)	1 (0.3–2.2)
S100 A9, µg/mL After	0.72 (0.22–0.75)	0.18 (0.05–0.39)*	0.54 (0.10–1.90)		

Values are medians (ranges).*p<0.05 vs before. ∧p = 0.063 vs before. **Bold:** significant difference in acute smoke response between two groups. #p = 0.056 vs young non-susceptible subjects. Old healthy smokers and never-smokers did not perform smoking experiments.

Due to experimental issues no quantifiable results were obtained for S100A8 (**Table S6 in File S1**); regarding ALDH3A1 no statistically significant differences between the groups were observed (**Table S7 in File S1**).

#### Immunohistochemistry confirmation: ALDH3A1

**Figure 3:**
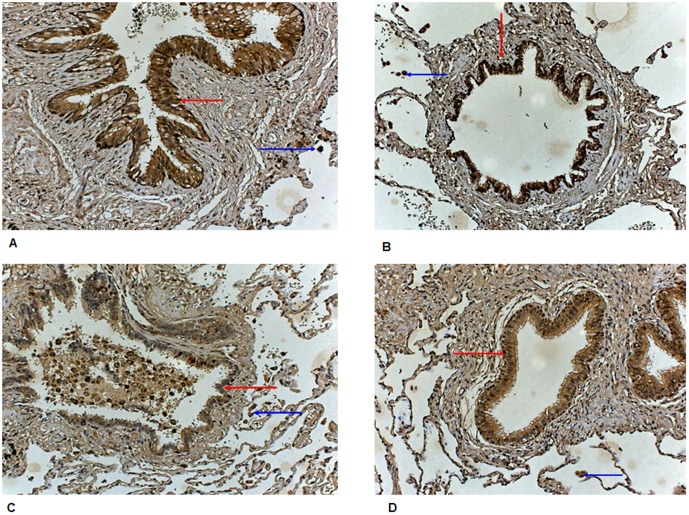
Immunohistochemistry of aldehyde dehydrogenase 3A1. Panel A: immunostaining of a COPD patient current smoker. Panel B: immunostaining of a healthy control current smoker. Panel C: immunostaining of a COPD patient ex-smoker. Panel D: immunostaining of a healthy control non-smoker. All COPD patients are GOLD STAGE II. The red arrows indicate epithelial cells and blue arrows indicate macrophages, more or less positive for ALDH3A1.

A semi-quantitative analysis was performed in a blinded fashion (by authors LF and ML) on ALDH3A1 expression in lung resection material of 5 COPD patients (current smokers), 5 COPD patients (ex-smokers), 5 healthy controls (current smokers), and 5 healthy never/ex-smokers (**Table S5 in File S1**). ALDH3A1 protein expression was clearly associated with smoking status (**Figure S5 in File S1**). Highest expression of ALDH3A1 was observed in macrophages and epithelial cells of COPD patients (current smokers), followed by healthy subjects (current smokers), and COPD patients (former smokers). The lowest expression was seen in healthy individuals and never smokers (**Figure 3**).

## Discussion

This is the first study to apply an unbiased proteomic approach to better understand the mechanisms underlying the development of COPD. iTRAQ analysis of ELF after acute smoke exposure demonstrated (in duplo) 9 proteins to be increased or decreased in young susceptible individuals, 4 proteins in the young non-susceptible individuals, and 3 in COPD patients. Six proteins were selected based on significant up- or down-regulation in two iTRAQ experiments, identification and quantification with two or more statistically significant peptides, and a biological function that might be implicated in the onset and progression of COPD. Of interest, two proteins (SerpinB3, Uteroglobin) decreased after smoking of 3 cigarettes in young non-susceptible individuals while remaining stable in young susceptible individuals. Four proteins (Peroxiredoxin I, S100A9, S100A8, ALDH3A1) decreased both in young susceptible and non-susceptible individuals. Peroxiredoxin, SerpinB3 and ALDH3A1 increased in COPD patients after a comparable smoke exposure. These differentially expressed proteins may play a role in protection against oxidative stress, anti-inflammatory responses and metabolizing toxic compounds, thus constituting plausible candidates involved in COPD development.

What might be the function of the above described differential proteins in relation to COPD more specifically? SerpinB3 inhibits several types of proteases and plays a role in modulating inflammation, programmed cell death and fibrosis [27]. S100A8 and S100A9 proteins, so called calgranulins, are known for their antimicrobial activity and their role as pro-inflammatory mediators in acute and chronic inflammation [28]–[30]. Uteroglobin may play a role in reducing airway inflammation and protecting against oxidative stress, in addition to its immunosuppressive and anti-tumor qualities [31]. Peroxiredoxins are known to control the response to oxidants and to play an anti-inflammatory role [32]. They are highly expressed in the healthy lung [28], and constitute a powerful defence against oxidative stress by decomposing peroxides, one of the major components of the tar phase of cigarette smoke. Finally, ALDH3A1 is one of the aldehyde dehydrogenases involved in the detoxification of carcinogenic aldehydes associated with cigarette smoke [33].

We found four proteins to decrease upon acute smoking irrespective of COPD susceptibility and hypothesize that these proteins play a role in orchestrating the normal inflammatory response to smoke exposure. In contrast, SerpinB3 and Uteroglobin decreased exclusively in young non-susceptible individuals, and ELISA experiments confirmed this for SerpinB3. The differential SerpinB3 and Uteroglobin response on smoking between the two young groups suggests that these proteins might be crucial for the very first steps towards COPD, given its modulatory function in inflammation and fibrosis [27] and release of lysosomal proteinases from damaged epithelial cells [34]. SerpinB3 concentrations have been shown to be higher in bronchoalveolar lavage fluid of smokers than non-smokers [35]. It was therefore an unanticipated observation that the expression of this protective protein was not restored to baseline 24 hours after acute smoke exposure in non-susceptible individuals, in contrast to the susceptible individuals. Whether this finding in ELF is a negative mirror of what occurs in the airway wall after an attack of cigarette smoking needs to be determined in further studies. In that case a lower value in ELF in non-susceptible youngsters indicates an increased use in the lung tissue, whereas this does not occur in susceptible individuals. Uteroglobin or human Clara cell protein (CC16) is a 15.8-kDa homodimeric protein secreted in large amounts into the airways by the non-ciliated bronchiolar Clara cells. The exact physiological function in the lung is not known, but it likely plays a role in reducing airway inflammation and protecting against oxidative stress, in addition to immunosuppressive and anti-tumor qualities [31]. In an acute smoke model in rats a dose dependent increase in serum Uteroglobin was demonstrated with a peak level 2 hours after smoking and a return to normal levels after 24 hours [36]. Our results show a decrease of Uteroglobin only in young non-susceptible individuals 24 hours after smoking. Unfortunately, we have no information about its presence immediately after smoking, so future studies, using less invasive sampling techniques, are needed to understand its complete time-response. COPD patients demonstrated an opposite response to acute smoking compared with young susceptible and non-susceptible individuals, with higher expression of Peroxiredoxin I, SerpinB3, and ALDH3A1 after smoking. This finding supports our choice of studying young individuals for better understanding of the very first steps of COPD induction. Apparently, the bronchial tree in COPD patients has changed dramatically after many years of smoking and is able to up-regulate these mainly protective proteins for at least 24 hours after smoke inhalation.

To assess if the detected proteins in COPD reflect a nonspecific response to chronic smoking or rather are a disease-specific characteristic we compared healthy smokers and never-smokers (at baseline). The iTRAQ and immunohistochemistry results of ALDH3A1 clearly show that this protective protein is strongly up-regulated due to chronic smoking both in COPD and healthy smokers. Interestingly, one proteomic study demonstrated increased levels in BAL fluid from ex-smoking COPD patients [8]. Regarding Uteroglobin we expected to find a smoking-induced reduction as chronic smoking has been associated with a lower number of Clara cells in the bronchial tree [37] as well as with lower levels in BAL fluid [38]–[41]. Moreover, reduced Uteroglobin protein levels have been demonstrated in BAL [41] and serum [41], [42] of COPD patients, whereas severe COPD patients demonstrated lower levels in sputum than moderate COPD patients [43]. In line, 2 proteomic studies demonstrated decreased levels in BAL fluid of asymptomatic smokers [40] and in induced sputum of smokers and COPD patients [12]. Our ELISA results indeed demonstrated reduced levels in ELF of COPD patients; a finding that did not match with the iTRAQ results in healthy smokers and never smokers.

A possible weakness of our study is that susceptibility at young age to develop COPD was based on family history. On the other hand, this strategy has been used in previous studies as well and provided clues for a genetic component of the disease [19], [44]–[46]. A second limitation is that we included a relatively low number of participants, and the groups were not optimally balanced for age and gender, which poses questions regarding the generalization of the obtained results. Third, the iTRAQ samples of the different groups needed to be pooled which allowed only 5 comparisons. On the other hand ELISA was performed on individual samples from a larger group of participants and was not limited in the number of comparisons. Despite the above described methodological drawbacks, our study was able to show statistically significant differences, suggesting major changes. The observed differential proteomic profiles in susceptible and non-susceptible individuals open avenues for further biomarker development in larger studies.

In conclusion, we describe one of the first studies to assess proteins associated with susceptibility to develop COPD using an unbiased approach. We found statistically significant changes in expression of candidate proteins upon acute smoke exposure, by studying two young cohorts of individuals and a group of older COPD patients. Our data show that already at young age, subjects with a positive family history of COPD respond differently to cigarette smoke than those with a negative family history. Particularly SerpinB3 and Uteroglobin were found to be proteins that may play a role in the development of COPD.

## Supporting Information

File S1 **Contains Tables S1-S7 and Figures S1-S6.**
(DOCX)

Table Data S1 **Proteins and relative peptides identified and quantified with confidence>95%.** Each reporter ion area 114, 115, 116 and 117 represent the group of young non-susceptible after acute smoking; young non-susceptible at baseline, young susceptible after acute smoking and young susceptible at baseline, respectively. In the group of older subjects the area represent COPD patients after acute smoking; COPD at baseline; Healthy subjects never smokers; healthy subjects current smokers.
(XSLX)
