# Supplementary material for: Susceptibility to COPD: Differential Proteomic Profiling after Acute Smoking
Source: PLoS One. 2014 Jul 18;9(7):e102037. doi: 10.1371/journal.pone.0102037 (PMC4103835; doi:10.1371/journal.pone.0102037)

**Susceptibility to COPD:**

**differential proteomic profiling after acute smoking**

*Lorenza Franciosi¹, Dirkje S. Postma^2^, Natalia Govorukhina¹, Maarten van den Berge^2^, Peter L. Horvatovich^1^, Fabrizia Fusetti^3^, Bert Poolman^3^, Monique E. Lodewijk^4^, Wim Timens^4^, Rainer Bischoff¹, Nick H. T. ten Hacken^2*^*

¹ University of Groningen, Department of Pharmacy, Analytical Biochemistry, Groningen, The Netherlands

² University of Groningen, University Medical Center Groningen, Department of Pulmonary Diseases, Groningen Research Institute of Asthma and COPD (GRIAC), Groningen, The Netherlands

^3^Department of Biochemistry, University of Groningen, Netherlands Proteomics Centre, Groningen, The Netherlands

^4^ University of Groningen, University Medical Center Groningen, Department of pathology, Groningen Research Institute of Asthma and COPD (GRIAC), Groningen, The Netherlands

**Combined Supporting Information Table S1. Proteins identified in pooled ELF of young individuals (susceptible and non-susceptible) in the first and duplicate iTRAQ-4plex experiments**

|  | **Both experiments** |
| --- | --- |
|  | AK1Adenylatekinase1 |
|  | AKR1A1Alcoholdehydrogenase[NADP+] |
|  | ALDH1A1cDNAFLJ50286,highlysimilartoRetinaldehydrogenase1 |
|  | ALDH3A1Aldehydedehydrogenase3A1 |
|  | ANXA1AnnexinA1 |
|  | ANXA536kDaprotein |
|  | ARHGDIA26kDaprotein |
|  | AZGP1Zinc-alpha-2-glycoprotein |
|  | BASP1Isoform1ofBrainacidsolubleprotein |
|  | CAPSCalcyphosin |
|  | CALM1;CALM3;CALM221kDaprotein |
|  | CRIP1Cysteine-richprotein1 |
|  | CST3Cystatin-C |
|  | CSTBCystatin-B |
|  | CTSDCathepsinD |
|  | CFL1Cofilin-1 |
|  | Diazepambindinginhibitor,spliceform1G |
|  | Elongationfactor1-alpha |
|  | ENO1Isoformalpha-enolaseofAlpha-enolase |
|  | GAPDHGlyceraldehyde-3-phosphatedehydrogenase |
|  | GSTA2GlutathioneS-transferaseA2 |
|  | GSTP1PutativeuncharacterizedproteinGSTP1 |
|  | HDGFHepatoma-derivedgrowthfactor |
|  | Heatshock70kDaprotein1 |
|  | HMGB1HighmobilitygroupproteinB1 |
|  | HP42kDaprotein |
|  | IGHG1 |
|  | IGHA1 |
|  | IGLV2-14 |
|  | KRT1549kDaprotein |
|  | KRT8Keratin,typeIIcytoskeletal8 |
|  | Lactotransferrin |
|  | LCN2PutativeuncharacterizedproteinLCN2 |
|  | LUZP6PutativeuncharacterizedproteinMTPN |
|  | LYZLysozymeC |
|  | NQO1NAD(P)Hdehydrogenase,quinone1(NQO1) |
|  | PARK7ProteinDJ-1 |
|  | PDLIM1PDZandLIMdomainprotein1 |
|  | PEBP1Phosphatidylethanolamine- |
|  | bindingprotein1 |
|  | PFN1Profilin-1 |
|  | PPIAPeptidyl-prolylcis-transisomeraseA |
|  | PRDX1Peroxiredoxin-1 |
|  | PRDX5Peroxiredoxin-5 |
|  | PRSS128kDaprotein |
|  | PSME1proteasomeactivatorcomplexsubunit1isoform2 |
|  | S100A11ProteinS100-A11 |
|  | S100A6ProteinS100-A6 |
|  | S100A8ProteinS100-A8 |
|  | S100A9ProteinS100-A9 |
|  | S100PProteinS100-P |
|  | Serumalbumin |
|  | SCGB1A1Uteroglobin |
|  | SELENBP1Isoform1ofSelenium-bindingprotein1 |
|  | SERPINB1Leukocyteelastaseinhibitor |
|  | SERPINB3Isoform1ofSerpinB3 |
|  | SH3BGRLSH3domain-bindingglutamicacid-rich-likeprotein |
|  | SOD1Superoxidedismutase[Cu-Zn] |
|  | TAGLN224kDaprotein |
|  | TALDO1Transaldolase |
|  | TFSerotransferrin |
|  | TXNThioredoxin,isoformCRA_b |
|  | UBB;RPS27A;UBCUbiquitin |
|  | YWHAZ14-3-3proteinzeta/delta |
|  | **First experiment** |
|  | 21kDaprotein |
|  | ACTB14kDaprotein |
|  | CST1Cystatin-SN |
|  | CTSCDipeptidylpeptidase1 |
|  | EIF4HSimilartomKIAA0038protein |
|  | ENSAIsoform4ofAlpha-endosulfine |
|  | Glutathionreductasedelta8+9alternativesplicingvariant |
|  | GOLM1Isoform1ofGolgimembraneprotein1 |
|  | HBA1;HBA2Hemoglobinalpha-2 |
|  | Hematologicalandneurologicalexpressed1-likeprotein |
|  | HNRNPA2B1 |
|  | IGHA2 |
|  | IGJPutativeuncharacterizedproteinIGJ |
|  | IGKCIgkappachainCregion |
|  | KRT10Keratin,typeIcytoskeletal10 |
|  | KRT1Keratin,typeIIcytoskeletal1 |
|  | LASP1PutativeuncharacterizedproteinLASP1 |
|  | LCN1L1Putativelipocalin1-likeprotein1 |
|  | LOC100126583;IGHA2 |
|  | LOC100290309hypotheticalproteinXP_002348012 |
|  | LOC389842similartoRANbindingprotein1 |
|  | LTFcDNAFLJ58679,highlysimilartoLactotransferrin |
|  | MB12kDaprotein |
|  | MYL12Bmyosinregulatorylightchain12BisoformB |
|  | Nuclearubiquitouscaseinandcyclin-dependentkinasessubstrate |
|  | ORM1orosomucoid1precursor |
|  | PIGRPolymericimmunoglobulinreceptor |
|  | PRDX6Peroxiredoxin-6 |
|  | PTMAPutativeuncharacterizedproteinPTMA |
|  | SERPINA1Isoform3ofAlpha-1-antitrypsin |
|  | STIP1Stress-induced-phosphoprotein1 |
|  | SUMO3cDNAFLJ57440,Smallubiquitin-relatedmodifier3 |
|  | TIMP1TIMPmetallopeptidaseinhibitor1 |
|  | TPI1;TPI1P1triosephosphateisomerase1isoform2 |
|  | TPM3PutativeuncharacterizedproteinDKFZp686J1372 |
|  | TPPP3Tubulinpolymerization-promotingproteinfamilymember3 |
|  | TTR20kDaprotein |
|  | TXNDC179kDaprotein |
|  | YWHAE22kDaprotein |
|  | ZG16BZymogengranuleprotein16homologB |
|  | **Second Experiment** |
|  | ACTG1Actin,cytoplasmic2 |
|  | ADH1CAlcoholdehydrogenase1C |
|  | ALDOA45kDaprotein |
|  | Alpha-amylase1 |
|  | ANXA2Isoform1ofAnnexinA2 |
|  | B2MBeta-2-microglobulin |
|  | C6orf58UPF0762proteinC6orf58 |
|  | CASTIsoform6ofCalpastatin |
|  | CST4Cystatin-S |
|  | CST5Cystatin-D |
|  | DEFA3Neutrophildefensin3 |
|  | DSTNDestrin |
|  | EZR69kDaprotein |
|  | \| FTH1Ferritinheavychain \| \| --- \| |
|  | GSNcDNAFLJ53327,highlysimilartoGelsolin |
|  | HBDBeta-globingenefromathalassemiapatient |
|  | HeatshockproteinHSP90-alpha |
|  | HeatshockproteinHSP90-beta |
|  | HEBP2HEBP2protein(Fragment) |
|  | HINT1Histidinetriadnucleotide-bindingprotein1 |
|  | HNRNPDIsoform3ofHeterogeneousnuclearribonucleoproteinD0 |
|  | IGHG2 |
|  | IGJImmunoglobulinJchain |
|  | IGLV2-14 |
|  | KRT9Keratin,typeIcytoskeletal9 |
|  | LGALS3Galectin-3 |
|  | Longpalate,lungandnasalepitheliumcarcinoma-associatedprotein1 |
|  | MSMBIsoformPSP94ofBeta-microseminoprotein |
|  | NCLNucleolin |
|  | NEDD89kDaprotein |
|  | PGK1Phosphoglyceratekinase1 |
|  | PIPProlactin-inducibleprotein |
|  | Polymericimmunoglobulinreceptor |
|  | PRDX2Peroxiredoxin-2 |
|  | PRDX6Peroxiredoxin-6 |
|  | Pre-Blymphocytegene2 |
|  | PSAPIsoformSap-mu-6ofProactivatorpolypeptide |
|  | PSMA6Proteasomesubunitalphatype-6 |
|  | PTMAPutativeuncharacterizedproteinPTMA |
|  | Putativeuncharacterizedprotein |
|  | RibosomalproteinL29(RPL29),mRNA |
|  | SLPIAntileukoproteinase |
|  | SUMO4Smallubiquitin-relatedmodifier4 |
|  | TFF3trefoilfactor3precursor |
|  | TMSL1Putativethymosinbeta-4-likeprotein1 |
|  | TPM3tropomyosinalpha-3chainisoform4 |
|  | triosephosphateisomerase1isoform2 |
|  | TUBA1CTUBA1Cprotein |
|  | TUBB2CTubulinbeta-2Cchain |
|  | Tubulinpolymerization-promotingproteinfamilymember3 |
|  | UBE2IUbiquitincarrierprotein |
|  | WFDC2Isoform1ofWAPfour-disulfidecoredomainprotein2 |
|  | YWHAE14-3-3proteinepsilon |

In the group of young subjects totally 157 proteins were identified in two experiments, 64 of them were overlapping in both experiments.

**Table S2. Proteins identified in pooled ELF of the old individuals (COPD patients, healthy smokers and non-smokers) in the first and duplicate iTRAQ-4plex experiments**

|  | **Both experiments** |
| --- | --- |
|  | AK1Adenylatekinaseisoenzyme1 |
|  | AKR1A1Alcoholdehydrogenase[NADP+] |
|  | ALDH1A1Retinaldehydrogenase1 |
|  | ALDH3A1Aldehydedehydrogenase3A1 |
|  | ALDOA45kDaprotein |
|  | ANXA1AnnexinA1 |
|  | ANXA536kDaprotein |
|  | AZGP1Zinc-alpha-2-glycoprotein |
|  | B2McDNAFLJ57067,highlysimilartoBeta-2-microglobulin |
|  | C6orf58UPF0762proteinC6orf58 |
|  | CALM1;CALM3;CALM221kDaprotein |
|  | CAPSCalcyphosin |
|  | CBR1Carbonylreductase[NADPH]1 |
|  | CFL1Cofilin-1 |
|  | CRIP1Cysteine-richprotein1 |
|  | CST3Cystatin-C |
|  | CSTBCystatin-B |
|  | CTSDCathepsinD |
|  | Diazepambindinginhibitor,spliceform1G |
|  | DMBT1Isoform8ofDeletedinmalignantbraintumors1protein |
|  | ENO1Isoformalpha-enolaseofAlpha-enolase |
|  | FAM3DProteinFAM3D |
|  | GAPDHGlyceraldehyde-3-phosphatedehydrogenase |
|  | GSTP1PutativeuncharacterizedproteinGSTP1 |
|  | HBA1;HBA2Hemoglobinsubunitalpha |
|  | Hematologicalandneurologicalexpressed1-likeprotein |
|  | HMGB1HighmobilitygroupproteinB1 |
|  | HPXHemopexin |
|  | IGHA1 |
|  | IGHG1 |
|  | IGHG2 |
|  | IGLV2-14 |
|  | KRT1Keratin,typeIIcytoskeletal1 |
|  | Lactotransferrin |
|  | LASP1Isoform1ofLIMandSH3domainprotein1 |
|  | LCN1Lipocalin-1 |
|  | LCN2Lipocalin2 |
|  | LGALS3Galectin-3 |
|  | LYZLysozymeC |
|  | NQO1NAD(P)Hdehydrogenase[quinone]1isoformb |
|  | PARK7ProteinDJ-1 |
|  | PEBP1Phosphatidylethanolamine-bindingprotein1 |
|  | PFN1Profilin-1 |
|  | PIGRPolymericimmunoglobulinreceptor |
|  | PPIAPeptidyl-prolylcis-transisomeraseA |
|  | PRDX1Peroxiredoxin-1 |
|  | PRDX2Peroxiredoxin-2 |
|  | PRDX5Peroxiredoxin-5 |
|  | PRDX6Peroxiredoxin-6 |
|  | PRSS128kDaprotein |
|  | PSAPIsoformSap-mu-6ofProactivatorpolypeptide |
|  | PTMAPutativeuncharacterizedproteinPTMA |
|  | Putativeuncharacterizedprotein |
|  | S100A11ProteinS100-A11 |
|  | S100A6ProteinS100-A6 |
|  | S100A8ProteinS100-A8 |
|  | S100A9ProteinS100-A9 |
|  | S100PProteinS100-P |
|  | SCGB1A1Uteroglobin |
|  | SELENBP1Isoform1ofSelenium-bindingprotein1 |
|  | SERPINB3Isoform1ofSerpinB3 |
|  | Serumalbumin |
|  | SH3BGRLSH3domain-bindingglutamicacid-rich-likeprotein |
|  | SOD1Superoxidedismutase[Cu-Zn] |
|  | SUMO3SMT3suppressorofmiftwo3homolog3(Yeast) |
|  | TAGLN224kDaprotein |
|  | TFSerotransferrin |
|  | TPPP3Tubulinpolymerization-promotingproteinfamilymember3 |
|  | TXNThioredoxin |
|  | YWHAZ14-3-3proteinzeta/delta |
|  | **First experiment** |
|  | Actin,alpha,cardiacmuscle(ACTC) |
|  | AKR1B10Aldo-ketoreductasefamily1memberB10 |
|  | Anti-(ED-B)scFV(Fragment) |
|  | BASP1Isoform1ofBrainacidsolubleprotein1 |
|  | C19orf33Isoform1ofImmortalizationup-regulatedprotein |
|  | CASTIsoform6ofCalpastatin |
|  | CLIC1Chlorideintracellularchannelprotein1 |
|  | CRISP3Cysteine-richsecretoryprotein3 |
|  | CSTAPutativeuncharacterizedproteinCSTA |
|  | EEF1A1;EEF1A1P5Putativeelongationfactor1-alpha-like3 |
|  | EIF4HSimilartomKIAA0038protein |
|  | EWSR1PutativeuncharacterizedproteinEWSR1 |
|  | GCIsoform2ofVitaminD-bindingprotein |
|  | GOLM1Isoform1ofGolgimembraneprotein1 |
|  | GSTA2GlutathioneS-transferaseA2 |
|  | HBBHemoglobinsubunitbeta |
|  | HDGFHepatoma-derivedgrowthfactor |
|  | HeterogeneousnuclearribonucleoproteinA1-like2 |
|  | HNRNPA2B1PutativeuncharacterizedproteinH |
|  | HP42kDaprotein |
|  | HSPA6Heatshock70kDaprotein6 |
|  | IGHA2 |
|  | IGHV3OR16-14hypotheticalproteinXP_002343513 |
|  | IGJPutativeuncharacterizedproteinIGJ |
|  | KRT10Keratin,typeIcytoskeletal10 |
|  | KRT1549kDaprotein |
|  | KRT9Keratin,typeIcytoskeletal9 |
|  | LTFcDNAFLJ58679,highlysimilartoLactotransferrin |
|  | NCLNucleolin |
|  | NRNPA2B1 |
|  | Nuclearubiquitouscaseinandcyclin-dependentkinasessubstrate |
|  | ORM1orosomucoid1precursor |
|  | PDLIM1PDZandLIMdomainprotein1 |
|  | PRR4proline-richprotein4isoform1 |
|  | RPS6RibosomalproteinS6,isoformCRA_a |
|  | S100A2proteinS100-A2 |
|  | SERPINA1Isoform1ofAlpha-1-antitrypsin |
|  | TIMP1TIMPmetallopeptidaseinhibitor1 |
|  | TMSB10Thymosinbeta-10 |
|  | TPI1;TPI1P1triosephosphateisomerase1isoform2 |
|  | Tubulin-specificchaperoneA |
|  | UBA52ubiquitinandribosomalproteinL40precursor |
|  | YWHAE22kDaprotein |
|  | **Second experiment** |
|  | ACTG1Actin,cytoplasmic2 |
|  | CATHEPSINB |
|  | cDNAFLJ59092 |
|  | CST1Cystatin-SN |
|  | DEFA3Neutrophildefensin3 |
|  | FTH1Ferritinheavychain |
|  | GSTA1GlutathioneS-transferaseA1 |
|  | GSTO1GlutathioneS-transferaseomega-1 |
|  | HBDBeta-globingenefromathalassemiapatient |
|  | DSTNdestrinisoformb |
|  | DYNLL1Dyneinlightchain1,cytoplasmic |
|  | Heatshock70kDaprotein1 |
|  | HNRNPDPutativeuncharacterizedproteinHNRNPD |
|  | IGJImmunoglobulinJchain |
|  | IGLV2-14 |
|  | KRT8Keratin,typeIIcytoskeletal8 |
|  | LCN1L1Putativelipocalin1-likeprotein1 |
|  | Longpalate,lungandnasalepitheliumcarcinoma-associatedprotein1 |
|  | MSMBIsoformPSP94ofBeta-microseminoprotein |
|  | ORM2Alpha-1-acidglycoprotein2 |
|  | PGK1Phosphoglyceratekinase |
|  | PSME1Proteasomeactivatorcomplexsubunit1 |
|  | RDXRadixin |
|  | RRBP1p180/ribosomereceptor |
|  | SLPIAntileukoproteinase |
|  | TALDO1Transaldolase |
|  | TFF3trefoilfactor3precursor |
|  | TMSL3Thymosinbeta-4-likeprotein3 |
|  | Triosephosphateisomerase1isoform2 |
|  | UBB;RPS27A;UBCUbiquitin |
|  | WFDC2Isoform1ofWAPfour-disulfidecoredomainp rotein2 |

In the group of old subjects totally 144 proteins were identified in two experiments, 70 of them were overlapping in both experiments.

**Table S3. Proteins differentially expressed at baseline**

| **Young susceptible higher than non-susceptible** | |
| --- | --- |
| FIRST  **Annexin A5**  **Proteasome activator complex subunit 1 isoform 2**  **Protein S100 A9**  **Protein S100 A8**  **Retinal dehydrogenase 1**  **Serpin B3**  14-3-3 protein zeta/delta  Anti-folate binding protein  Elongation factor 1-alpha  Glyceraldehyde-3-phosphate dehydrogenase  IGHA2  LOC100290309 hypothetical protein  MB 12 kDa protein  Myosin regulatory light chain 12B isoform B  NAD(P)H dehydrogenase  Heat shock protein 70  Protein LASP1  Serum albumin  TPM3 | SECOND  **Annexin A5**  **Proteasome activator complex subunit 1 isoform 2**  **Protein S100 A9**  **Protein S100 A8**  **Retinal dehydrogenase 1**  **Serpin B3**  Adenylate kinase 1  Alcohol dehydrogenase [NADP+]  Annexin A1  Diazepam binding inhibitor, splice form 1G  Heat shock protein 70  IGHA1  Keratin type II cytoskeletal 8  Lactotransferrin  Peroxiredoxin V  Protein S100 A11  Serotransferrin |
| **Young susceptible lower than non-susceptible** | |
| FIRST  Beta-2-microglobulin  Cystatin-SN  Glutathione S-transferase A2  Lactotransferrin  Lipocalin-1 | SECOND  Calcyphosin  Cystatin S  IGHG1  Serum Albumin |
| **Old healthy smoker higher than old never-smoker** | |
| FIRST  **ALDH3A1**  **AZGP1 (zinc-alpha-2 glycoprotein)**  **C6orf 58**  **CBR1 carbonyl reductase**  **Lactotransferrin**  **Lysozyme C**  **NQO1 NAD(P)H dehydrogenase quinone1**  Aldoketo reductase  GOLM1  MBT1  PRR4 proline-rich protein4  SOD1 superoxide dismutase | SECOND  **ALDH3A1**  **AZGP1**  **C6orf 58**  **CBR1 carbonyl reductase**  **Lactotransferrin**  **Lysozyme C**  **NQO1 NAD(P)H dehydrogenase quinone1**  IG alpha-1  IG J  IGLV2-14  MSMB (Beta-microsemoniprotein)  PIGR (polymeric immunoglobulin receptor)  PRDX1- PRDX2  SLP1 antileukoproteinase  TFF3 |
| **Old healthy smoker lower than old never-smoker** | |
| FIRST  HBB (hemoglobin subunit Beta)  S100 A6 | SECOND  GAPDH (Glyceraldehyde 3-phosphate dehydrogenase) |

In the group of young subjects totally 39 proteins were differentially expressed between young susceptible and non-susceptible individuals at baseline in two experiments; with 6 proteins showing overlap in both experiments. In the group of old subjects totally 23 proteins were differentially expressed in old healthy smokers and old never-smokers at baseline in two experiments; with 7 proteins showing overlap in both experiments. The proteins that overlap in both experiments are depicted in bold.

**Table S4. Proteins increased or decreased after acute smoke exposure**

| **Young susceptible increased** | |
| --- | --- |
| FIRST  **Cystatin-SN**  Lactotransferrin  Lipocalin 1  Unidentified protein (TGSGDIENNYND) | SECOND  **Cystatin-SN**  Alpha-amylase 1  Cystatin-D  IGHG1  Keratin 1 type II cytoskeletal 1  Serum albumin |
| **Young susceptible decreased** | |
| FIRST  **Aldehyde dehydrogenase 3A1**  **Protein S100 A9**  **Protein S100 A8**  **Annexin A1**  **Protein S100 P**  **NAD(P)H dehydrogenase quinone 1**  **Annexin A5**  **Retinal dehydrogenase ALDH1A1**  ACTB 14 kDa protein  Elongation factor 1-alpha  Glutathione S-transferase P 1  Glyceraldehyde 3-phosphate dehydrogenase  Heat shock protein 70  Keratin 1  Keratin 10  MYL12B myosin regulatory ligand  Proteasome activator PSME1  Unidentified protein (FLIDGFPR)  YWHAE 22 kDa protein | SECOND  **Aldehyde dehydrogenase 3A1**  **Protein S100 A9**  **Protein S100 A8**  **Annexin A1**  **Protein S100 P**  **NAD(P)H dehydrogenase quinone 1**  **Annexin A5**  **Retinal dehydrogenase ALDH1A1**  Alcohol Dehydrogenase 1C  Diazepam binding inhibitor  Galectin-3  Glutathione S-transferase A2  Peroxiredoxin I  Peroxiredoxin V  Profilin-1  Protein S100 A11  Triosephosphate isomerase 1 isorform 2 |
| **Young non-susceptible increased** | |
| FIRST  **Keratin 1 type II cytoskeletal 1**  Serum albumin  Unidentified protein (TGSGDIENYND) | SECOND  **Keratin, type II cytoskeletal 1**  Alcohol dehydrogenase [NADP+]  Alpha-amylase 1  BASP1 Isoform 1 of Brain soluble acid soluble protein 1  Cystatin-S  Cystatin-D HMGB1  High Mobility Group Protein B1  Keratin, type I cytoskeletal 9 |
| **Young non-susceptible decreased** | |
| FIRST  **Aldehyde dehydrogenase 3A1**  **Polymeric immunoglobulin receptor**  Alpha-enolase 1  Unidentified protein (EIVMTQSPATD) | SECOND  **Aldehyde dehydrogenase 3A1**  **Polymeric immunoglobulin receptor**  Actin, cytoplasmatic 2  ALDH1A1 Retinal Dehydrogenase  Aldolase A  Annexin A1  Calmodulin  Galectin-3  HBD Beta-globin  Heat shock protein 90-alpha  Heat shock protein 90-beta IGHA1  Lactotransferrin  Serotransferrin  Serum albumin  Peroxiredoxin I  Peptidyl-prolyl cis-trans isomerase A  PLUNC  Profilin-1  Protein S100 A8  Proterin S100 A9  Serpin B3  Triosephosphate isomerase 1 isoform 2  Uteroglobin |
| **COPD increased** | |
| FIRST  **Serum Albumin**  **Aldehyde dehydrogenase 3A1**  Calcyphosin  Lipocalin 1  Peroxiredoxin I  PTMA | SECOND  **Serum albumin**  **Aldehyde dehydrogenase 3A1**  Annexin A5  Carbonil Reductase [NADPH] 1  Glutathione S-transferase P  Heat shock protein 70  Radixin  Retinal Dehydrogenase ALDH1A1  Serpin B3 |
| **COPD decreased** | |
| FIRST  **Uteroglobin**  Keratin 1  Lactotransferrin  Lipocalin 2  Lysozyme C  GOLM1  IGLV2-14 | SECOND  **Uteroglobin**  Trefoil factor 3 precursor |

In the group of young susceptible subjects the expression of totally 37 proteins increased or decreased after smoking in 2 experiments, showing overlap of 9 proteins in both experiments. In the group of young non-suceptible individuals this was 35 and 3 proteins respectively, in COPD 21 and 3 proteins. The proteins that overlap in both experiments are depicted in bold.

**Table S5. Subject characteristics for immunohistochemistry of ALDH3A1 in lung tissue**

|  | **COPD**  **current smokers** | **COPD**  **Ex-smokers** | **non-COPD**  **current smokers** | **non-COPD**  **never/ex smokers** |
| --- | --- | --- | --- | --- |
| Female / Male | 0/5 | 0/5 | 2/3 | 2/3 |
| Age | 69  (44-71) | 72  (65-76) | 63  (51-68) | 50  (36-73) |
| Pack-years | 28  (14-50) | 30  (8-50) | 50  (25-75) | 0  (0-50) |

Values are medians (ranges), or numbers.

**Table S6. ELISA results of protein S100A8 of individual epithelial lining fluid (ELF) samples of young susceptible individuals, young non-susceptible individuals, healthy subjects and established COPD patients, before and after acute smoking. 5 Young susceptible, 5 Young non-susceptible, 7 COPD patients were investigated but only for few samples it was possible to determine the concentrations. Results are given in concentrations (ng/mL).**

| **Young non susceptible before** | **Young non susceptible after** | **Young susceptible before** | **Young susceptible after** | **COPD before** | **COPD after** | **Healthy non smokers** | **Healthy smokers** |
| --- | --- | --- | --- | --- | --- | --- | --- |
| 0.132 | NA | \| 0.02657 \| \| --- \| \| 0.08113 \| \| 0.0302 \| | 0.03384 | \| 8.38E-03 \| \| --- \| \| 0.01929 \| \| 1.11E-03 \| \| 8.38E-03 \| | 0.03384 | \| 8.38E-03 \| \| --- \| \| 1.11E-03 \| \| 0.143 \| \| 0.01929 \| \| 0.0302 \| \| 0.2484 \| \| 1.11E-03 \| \| 0.01929 \| \| 0.2121 \| \| 0.2375 \| \| 0.263 \| | \| 0.06658 \| \| --- \| \| 0.03748 \| \| 8.38E-03 \| \| 0.143 \| \| 0.01202 \| \| 0.02293 \| \| 0.07749 \| |

**Table S7. ELISA results of protein ALDH3A1 of individual epithelial lining fluid (ELF) samples of young susceptible individuals, young non-susceptible individuals, healthy subjects and established COPD patients, before and after acute smoking. No significant differences were found comparing the different groups (p value >0.05). Results are given in concentrations (ng/mL).**

| **Young non susceptible before** | **Young non susceptible after** | **Young susceptible before** | **Young susceptible after** | **COPD before** | **COPD after** | **Healthy non smokers** | **Healthy smokers** |
| --- | --- | --- | --- | --- | --- | --- | --- |
| \| 0.05029 \| \| --- \| \| 0.05457 \| \| 0.05674 \| \| 0.0461 \| \| 0.05242 \| | \| 0.04201 \| \| --- \| \| 0.06565 \| \| 0.05029 \| \| 0.06339 \| \| 0.04404 \| | \| 0.05242 \| \| --- \| \| 0.06115 \| \| 0.05894 \| \| 0.07254 \| \| 0.05457 \| | \| 0.05674 \| \| --- \| \| 0.05894 \| \| 0.06115 \| \| 0.07488 \| \| 0.05674 \| | \| 0.04404 \| \| --- \| \| 0.06339 \| \| 0.05457 \| \| 0.07022 \| \| 0.08929 \| \| 0.06565 \| \| 0.05242 \| | \| 0.1018 \| \| --- \| \| 0.082 \| \| 0.07488 \| \| 0.05674 \| \| 0.04819 \| \| 0.08441 \| \| 0.05894 \| | \| 0.06115 \| \| --- \| \| 0.06792 \| \| 0.07022 \| \| 0.07254 \| \| 0.04819 \| \| 0.04819 \| \| 0.05674 \| \| 0.04819 \| \| 0.07022 \| \| 0.07488 \| \| 0.06339 \| \| 0.04819 \| \| 0.07488 \| | \| 0.04404 \| \| --- \| \| 0.05457 \| \| 0.1147 \| \| 0.05894 \| \| 0.05894 \| \| 0.07022 \| \| 0.06339 \| \| 0.05029 \| \| 0.04819 \| |

**Table Data S1. Proteins and relative peptides identified and quantified with confidence>95%. Each reporter ion area 114, 115, 116 and 117 represent the group of young non-susceptible after acute smoking; young non-susceptible at baseline, young susceptible after acute smoking and young susceptible at baseline, respectively. In the group of older subjects the area represent COPD patients after acute smoking; COPD at baseline; Healthy subjects never smokers; healthy subjects current smokers**.

**This table is uploaded separately as excel file.**

**Figure S1:**

**Main steps of statistical analysis to identify discriminative compounds from iTRAQ analysis data. The first step consists of calculating a histogram of natural logarithm reporter ions ratios of fragmented peptides between -4.6 (corresponding to ratio 0.01) and 2.3026 (corresponding to ratio 10) with step of 0.05. In the second step Savitzky-Golay smoothing with degree of 1 is applied. In the third step, Gaussian curve of normal distribution is fitted to the smoothed histogram. The parameters of the fitted curve were used to calculate the standard deviation (SD). The subplot for step 3 shows the data points of the smoothed curve of step 2 with blue dots, while the fitted Gaussian curve is shown with continuous red curve. For Gaussian curve fitting natural logarithm of ratios between -1 and 1 were used. Lower and upper threshold is selected at ±2.5 SD. The natural logarithm ratios of the reporter ions (in this example 114 and 115) of the selected discriminatory peptides with corresponding protein name are plotted in step 4. The method suppose that the majority of the protein do not change between the pooled samples, and therefore the central part of the Gaussian distribution can be used to fit a theoretical Gaussian curve. The peptides of discriminatory proteins are in the tails of the Gaussian. Gaussian curve reflect normal distribution of natural logarithm of reporter ion ratios and this curve can be used to setup a threshold based on type I error to select discriminatory peptides, and finally the list of discriminatory proteins**.

**Figure S2:**

**Overlap of the identified proteins between the first and second iTRAQ experiment in the young subjects (susceptible and non-susceptible) and the old subjects (COPD and healthy controls)**

**Figure S3:**

**Overlap of the differentially expressed proteins in young subjects (both susceptible vs non-susceptible) and old subjects (old healthy smokers vs old never smokers) at baseline**

**
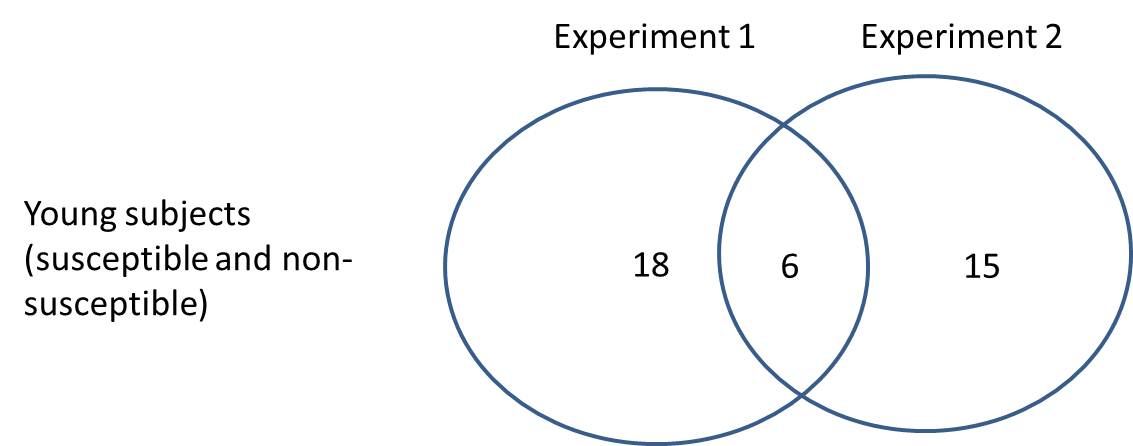
**


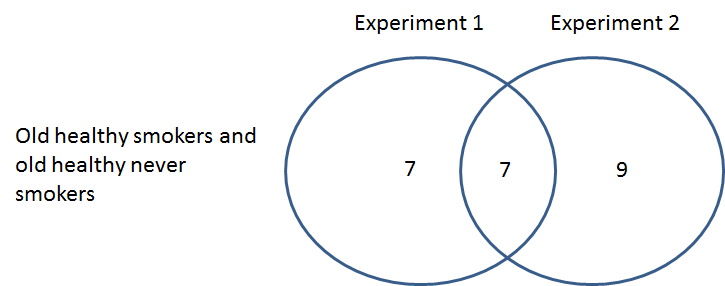


**Figure S4:**

**Overlap between the first and second iTRAQ experiment in differentially expressed proteins before and after smoking in the young susceptible subjects, young non-susceptible and the COPD patients**


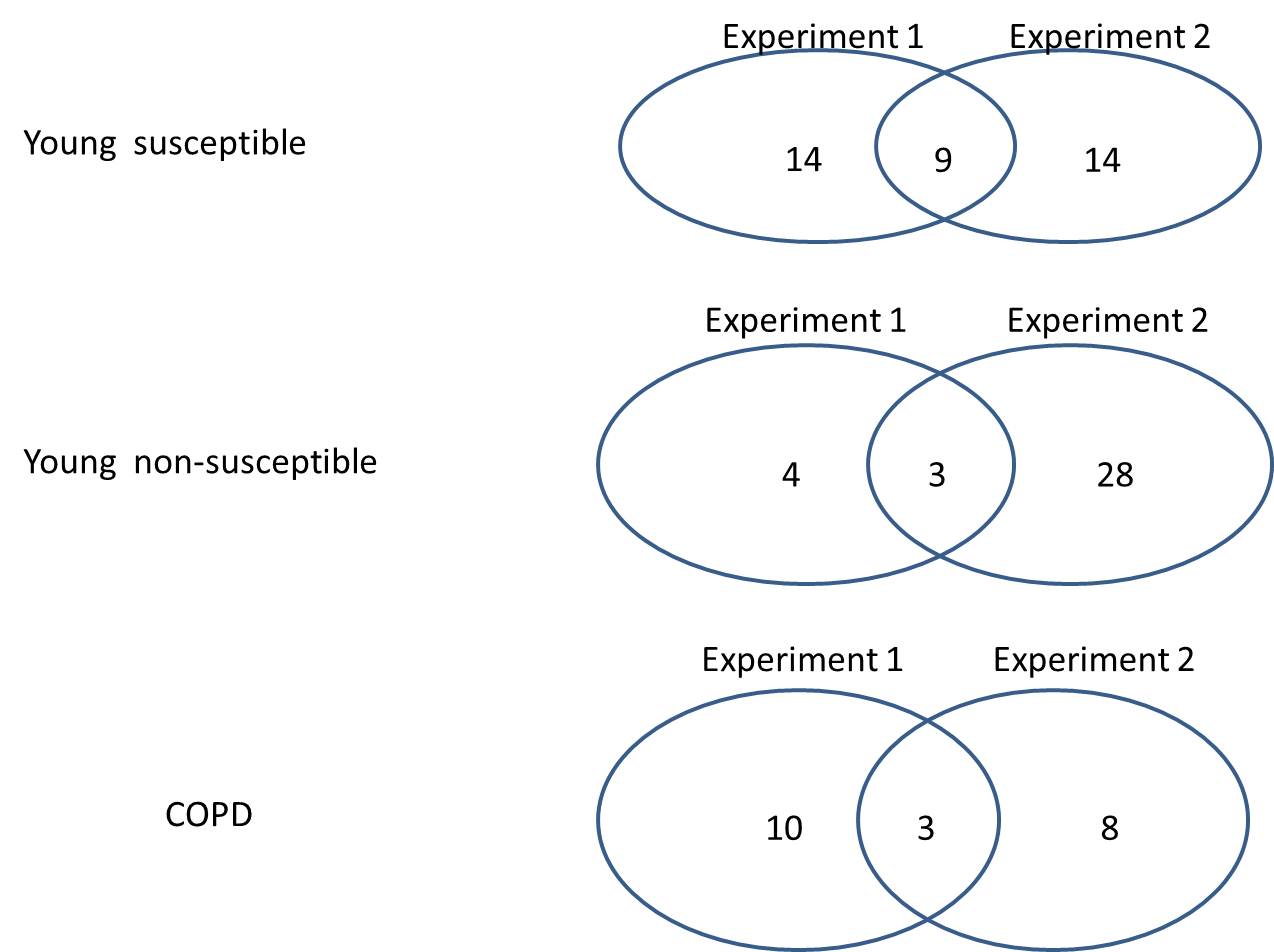


**Figure S5:**

**Semiquantitative analysis from immunohistochemistry of aldehyde dehydrogenase 3A1**


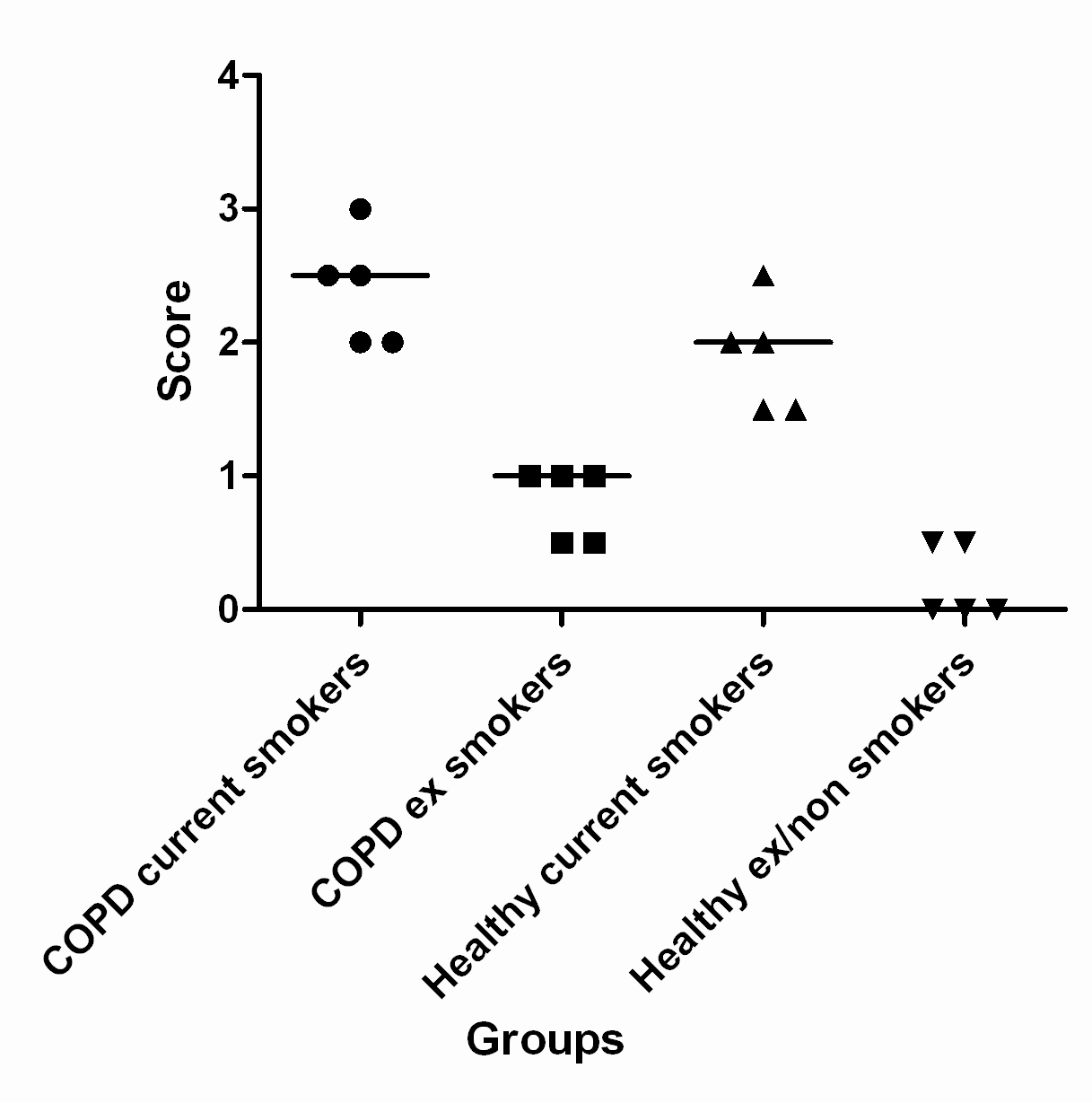


Staining scoring: 0= negative; 1= weak; 2= positive; 3= strong positive

**Figure S6:**

**MS/MS spectra of peptide IGHPAPNFK of Peroxiredon I (precursor 1268.71). Peaks at 114, 115, 116 and 117 represent the group of young non-susceptible after acute smoking; young non-susceptible at baseline, young susceptible after acute smoking and young susceptible at baseline, respectively**.


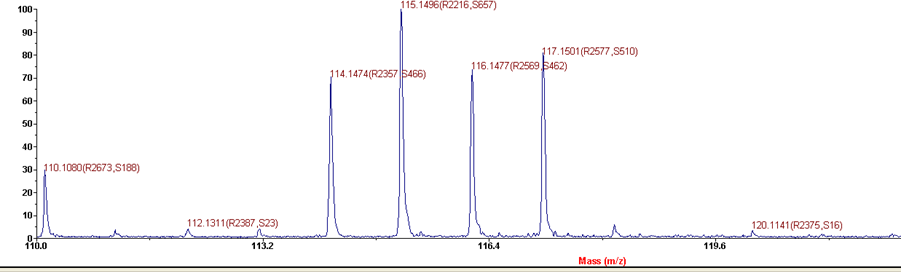

Supplement: File S1 — Contains Tables S1-S7 and Figures S1-S6. (DOCX) [file pone.0102037.s001.docx]
